# Articular surface mounted navigated total knee arthroplasty improves the reliability of component alignment

**DOI:** 10.1007/s00167-017-4433-x

**Published:** 2017-02-16

**Authors:** N. D. Clement, D. MacDonald, A. G. Burgess, C. R. Howie

**Affiliations:** 1 Department of Orthopaedics and Trauma, The Royal Infirmary of Edinburgh, Little France, Edinburgh, EH16 4SA UK; 2 University of Edinburgh, Edinburgh, UK

**Keywords:** Total knee arthroplasty, Navigation, Outcome, Alignment, Oxford knee score

## Abstract

**Purpose:**

The primary aim was to compare the early knee-specific functional outcome after articular surface mounted (ASM) navigation with non-navigated TKA. The secondary aims were to compare general physical and mental health improvement, patient satisfaction, and reliability of component alignment in the sagittal and coronal planes between ASM navigated TKA with that of non-navigated TKA.

**Methods:**

Prospective functional outcome and radiographic data were collect for 123 patients undergoing ASM navigation and 172 patients undergoing non-navigated TKA by a high volume single surgeon. Pre-operative and one-year Oxford knee score (OKS) and short form (SF-) 12 scores were collected. Patient satisfaction was also assessed at one year. Implant position was assessed on post-operative radiographs (alpha, beta, gamma, and sigma angles) by a blinded observer.

**Results:**

There was no significant difference for improvement in OKS, SF-12 physical or mental components, or satisfaction between the groups one year following surgery. The non-navigation group was significantly more likely to have outliers (greater than 3 degrees) in femoral varus/valgus coronal alignment [odds ratio (OR) 4.5, 95% confidence interval (CI) 1.0–20.7, *p* = 0.049] and for posterior tibial slope (OR 8.3, 95% CI 1.1–65.0, *p* = 0.03).

**Conclusions:**

ASM navigation significantly reduces the number of outliers for the femoral and tibial components when compared to conventional non-navigation alignment. However, the short-term functional outcome is not influenced by the surgical technique used. If the surgeon wants to reduce their number of outliers, then ASM navigation should be considered but the overall functional outcome in the short term is not influenced.

**Level of evidence:**

III Therapeutic investigation, retrospective cohort study.

## Introduction

Approximately, one in five patients are not satisfied with their total knee arthroplasty (TKA) [1], and one reason may be implant malalignment during surgery that could result in early revision for instability [2]. Over the last 20 years, there have been numerous publications supporting the increased accuracy of computer navigation over conventional instrumentation for TKA [3–5], however the uptake of this technology is not widespread [6]. Several level one studies have demonstrated that navigation for TKA results in less deviation from the mechanical axis, being more likely to be within three degrees of the planned coronal alignment [7–9]. It is also interesting to note that one of the most successful litigations after TKA is for technical errors such as malalignment (71%) [10].

Navigated TKA does have specific complications associated with the use of tracker pins. There have been several reports of peri-prosthetic fractures from the tracker pin sites [11, 12]. There has also been concerns regarding tracker movement during surgery especially in osteoporotic bone [13] and with use of a single pin [14]. Stability is most reliable when three pins are used in the metaphysis [15]; this may, however, increase the fracture risk from numerous cortical perforations. An articular surface mounted (ASM) tracker avoids any cortical deficits in the metaphysis and affords greater stability using four pins to secure the tracker placement. The authors are only aware of three published studies in the literature reporting the outcome of ASM navigation for TKA [15–17]. A randomised controlled trial by Harvie et al. [18] demonstrated ASM navigation to be as accurate as full navigation and that the operative time was significantly reduced. A retrospective comparative study illustrated the femoral component alignment to be more accurate with ASM navigation compared to conventional intramedullary instrumentation [19]. Despite this increased accuracy of the femoral component with ASM navigation, the functional outcome of the TKA is no different to that of a conventional intramedullary instrumentation [20].

The primary aim of this study was to compare the early knee-specific functional outcome after ASM navigation with non-navigated TKA. The secondary aims were to compare general physical and mental health improvement, patient satisfaction, and reliability of component alignment between ASM navigated TKA with that of non-navigated TKA. The hypothesis of the study was that navigation offers greater accuracy of component alignment with improved functional outcomes when compared to non-navigated TKA.

## Materials and methods

A retrospective cohort study was conducted. During a seven-year period (2007–2013), patients undergoing a TKA at the study centre by the senior author (CRH) had outcome data recorded prospectively. Inclusion criterion for this study were: primary osteoarthritis, no extra-articular deformity or reason why an intramedullary jig could not be used. Patients undergoing consecutive bilateral TKAs during the study period only had outcome and radiographic measures assessed for their first knee.

The patient demographics, ASA grade, body mass index (BMI) and patient reported outcome measures were recorded at the pre-operative assessment clinic. Oxford knee score (OKS) [21] and the short form (SF-) 12 score [22] were recorded pre-operatively and at one year post-operatively. The OKS consists of twelve questions assessed on a Likert scale with values from 0 to 4, a summative score is then calculated where 48 is the best possible score (least symptomatic) and 0 is the worst possible score (most symptomatic). The SF-12 is a generic assessment tool to measure a patients wellbeing, which is assessed using a physical component summary (PCS) and a mental component summary (MCS) [22]. Both the SF-12 PCS and MCS range from 0% (worst level of functioning) to 100% (best level of functioning). Patient satisfaction was assessed by asking the question “How satisfied are you with your operated knee?” 1 year after surgery. The response was recorded using a four-point Likert scale: very satisfied, satisfied, uncertain, and unsatisfied. Patients who recorded very satisfied or satisfied were classified as satisfied. This has been used previously to assess patient satisfaction after TKA [1].

**Fig. 1:**
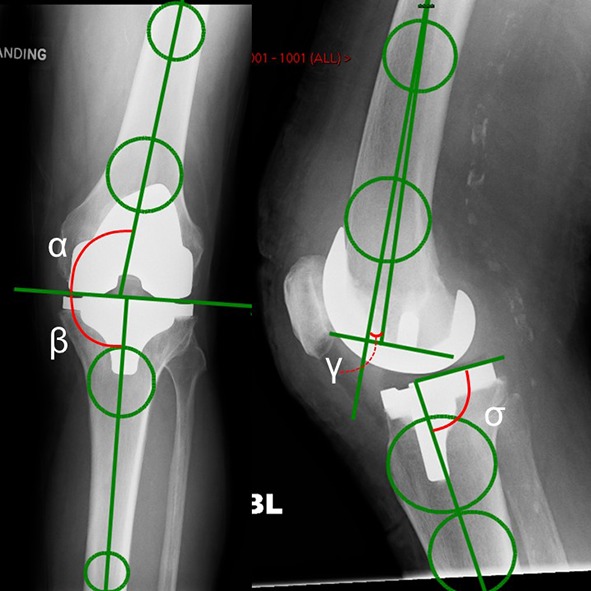
Anterior-posterior and lateral radiograph of the knee post operatively illustrating the angles measured (*α, β, γ*, and *σ*)

Radiographic assessment was performed using standard weight bearing anterior–posterior and lateral radiographs. The included patients did not have any extra-articular deformity and there was no clinical need to obtain a hip knee ankle (HKA) radiograph, which is the gold standard for measuring alignment. The circle method described by Veljkovic et al. [23] and validated for use around the knee by Zampogna et al. [24] was used to assess implant alignment. Alpha, beta, gamma, and sigma angles, as described by Shah et al. [19], were measured using digital radiographs (Kodac^©^ picture archiving and communication system on a liquid crystal display) and the graphic measuring tools available to one decimal place (Fig. 1).

There was a randomised controlled trial conducted at the study centre from 2008 to 2010 and patients recruited to this were removed from the presented cohort [25]. During the study period, the senior author performed or scrub-supervised all included TKAs. All patients underwent a cemented Triathlon (Stryker^®^) TKA using a measured resection technique. The technique (ASM navigation or conventional jig alignment) was assigned according to availability of surgical trays for patients who met the inclusion criterion. The primary technique was ASM navigation, but if the trays were not available for this conventional instrumentation was used. A mid-line medial para-patellar approach was made in all patients. ASM navigation was used for both femoral and tibial alignment; however, sizing of the femoral component and rotation (using Whiteside’s line [26]) was performed manually. The femoral component was aligned in 6° valgus and neutral flexion/extension (at the beginning of the series) and then 3° of flexion due to observed extension of some components, and zero varus/valgus with 3° posterior slope for the tibial component. Conventional jig alignment technique used intramedullary referencing for the femur and extramedullary for the tibia. The specified bone cuts were: 5° of valgus and neutral flexion/extension for the femoral component and zero varus/valgus with 3° of posterior slope for the tibial component. Length of operation (knife to skin to wound closure) was recorded from the hospital surgical database (Operating Room Scheduling Office System). All patients received three peri-operative doses of prophylactic antibiotics (cefuroxime). A standardised rehabilitation protocol as per local clinical care pathway was used for all patients, with active mobilisation on the first day post-operatively. Length of stay was recorded. Patients were then reviewed at 6 weeks, 6 and 12 months post-operatively.

Ethical approval was obtained from the regional ethics committee (Research Ethics Committee, South East Scotland Research Ethics Service, Scotland, 11/AL/0079) for collection, analysis, and publication of the presented data.

### Statistical analysis

Statistical Package for Social Sciences version 17.0 (SPSS Inc., Chicago, IL, USA) was used to analyse the data. Parametric and non-parametric tests were used as appropriate to assess continuous variables for significant differences between groups. A Student’s *t* test, unpaired and paired, or Mann Whitney U tests were used to compare linear variables between groups, and Pearson’s correlation was used to assess the relationship between linear variables. Dichotomous variables were assessed using a Chi-square test. Multivariable linear regression analyses were used to identify independent predictors of outcome (change in the OKS). A single measure intraclass correlation coefficient was used for the quantification of inter and intra observer reliability of the radiographic measurements. Values greater than 0.75 indicate satisfactory reliability [27]. A *p* value of <0.05 was defined as significant.

A post hoc power calculation was performed using the OKS (primary outcome measure), which has a defined minimal clinically important difference of 4 points [28] and a standard deviation (SD) of 10 points. This determined that 123 patients in the ASM group and 172 patients in the control group achieved a power of 0.92 using two-tailed analysis and an alpha of 0.05.

## Results

During this period, the senior author performed 398 TKAs of which 295 (74%) met the inclusion criteria and had pre-operative and post-operative outcome measures recorded. There were no significant differences between the groups (Table 1).

**Table 1:** Patient demographics and pre-operative functional scores according group

Demographic	Descriptive	Group		Odds ratio/difference	95% CI		*p* value
		ASM (*n* = 123)	Control (*n* = 172)		Lower	Upper	
Gender (M/F) (*n*, % of group)	Male	53	68	1.2	0.7	1.9	0.54*
	Female	70	104				
Mean age (years: mean, SD)		68.1 (10.3)	68.6 (10.4)	*0.5*	−1.9	2.9	0.67**
ASA grade (*n*, % of group)	I	14 (11.4)	12 (7.0)	–			0.96*
	II	103 (83.7)	152 (88.4)				
	III	6 (4.9)	8 (4.7)				
	IV	0	0				
	V	0	0				
BMI (mean, SD)		31.8 (6.5)	30.1 (4.9)	1.6	−2.1	5.4	0.37**
PROMs (mean, SD)							
OKS	Pre-operative	19.6 (7.6)	18.4 (8.5)	*1.2*	−0.7	3.1	0.21**
SF-12 PCS	Pre-operative	29.8 (8.0)	29.0 (8.4)	*0.8*	−1.1	2.7	0.73**
SF-12 MCS	Pre-operative	49.2 (12.2)	51.0 (11.2)	*2.1*	−1.0	4.4	0.16**

*ASM* articular surface mounted, *CI* confidence interval, *ASA* American Society of Anaesthesiologists, *BMI* body mass index, *PROMs* patient reported outcome measures, *OKS* Oxford knee score, *SF* short form, *PCS* physical component summary, *MCS* mental component summary

*Chi square test

**Unpaired *t* test

The inter-observer and intra-observer reliability of radiographic assignment of each of the radiographic measurements were reliable (Table 2). There was no significant difference in the radiographic measures between the groups (Table 3). The spread to the data was, however, much narrower in the ASM group which can be observed by the relatively smaller SD for each of the measurements. Patients in the control group were significantly more like to be outliers for femoral varus/valgus and tibial slope, with a trend towards significance for tibial varus/valgus compare to the ASM group (Table 4). In addition, when combining all outlier data, for any of the four angles measured, only 4% of patients in the ASM group were outliers compared to 11% of the control group (Table 4).

**Table 2:** Single measure intraclass correlation coefficients for inter- and intra- observer reliability for each of the radiographic measures used

Component	Plane	Intraclass correlation coefficient			
		Inter	95% CI	Intra	95% CI
Tibiofemoral angle		0.90	0.85–0.95	0.95	0.92–0.97
Femoral	Varus/valgus (alpha)	0.84	0.79–0.89	0.90	0.86–0.94
	Flexion/extension (gamma)	0.80	0.71–0.90	0.86	0.80–0.92
Tibial	Varus/Valgus (beta)	0.85	0.80–0.91	0.88	0.86–0.90
	Tibial slope (sigma)	0.81	0.77–0.85	0.85	0.80–0.90

*CI* confidence interval

**Table 3:** Radiographic measurements according to group

Component	Plane	Group (°)		*p* value*
		ASM (*n* = 123)	Control (*n* = 172)	
Tibiofemoral angle (mean, SD)		175.4 (2.0)	175.1 (3.1)	ns
Femoral (mean, SD)	Varus/valgus (alpha)	94.7 (1.5)	95.1 (3.0)	ns
	Flexion/extension (gamma)	0.4 (2.3)	3.2 (2.4)	ns
Tibial (mean, SD)	Varus/Valgus (beta)	89.9 (1.8)	89.8 (2.6)	ns
	Tibial slope (sigma)	3.2 (1.9)	3.3 (3.2)	ns

*ASM* articular surface mounted

*Unpaired *t* test

**Table 4:** Radiographic measurements illustrating outliers (greater than 3°) for each component and in combination according to group

Component	Plane	Group		Odds ratio	95% CI		*p* value*
		ASM (*n* = 123)	Control (*n* = 172)		Lower	Upper	
Femoral (*n*, % of group)	Varus/valgus	2 (1.6)	12 (7.0)	4.5	1.0	20.7	0.049
	Flexion/extension	3 (2.4)	5 (2.9)	1.1	0.3	5.1	ns
Tibial (*n*, % of group)	Varus/valgus	3 (2.4)	13 (7.6)	3.3	0.9	11.7	ns
	Tibial slope	1 (0.8)	11 (6.4)	8.3	1.1	65.0	0.03
Both (*n*, % of group)	Any	5 (4.1)	19 (11.0)	2.9	1.1	8.1	0.03

*ASM* articular surface mounted, *CI* confidence interval

*Chi-square test

There was no significant difference in the OKS or SF-12 scores at one year between the groups (Table 5). Seven patients did not complete their satisfaction rating at one year. The proportion of patients reporting satisfaction with their TKA at one year was 82.4% (98/119) for the ASM group and 80.5% (136/169) for the control group [odds ratio (OR) 1.1, 95% confidence interval (CI) 0.6–2.1, ns).

**Table 5:** Post-operative outcome measures and the difference relative to pre-operative scores according to group

Functional Measure	ASM (*n* = 123)		Control (*n* = 172)		Difference	95% CI	*p* value*
	Mean	SD	Mean	SD			
OKS							
Pre-operative	19.8	7.7	18.4	8.5	*1.2*	−0.7 to 3.1	ns
Post-operative	32.9	10.4	33.1	10.5	0.2	−2.2 to 2.7	ns
Difference	13.2	8.5	14.8	10.0	1.6	−0.4 to 3.6	ns
95% CI	11.6–14.7		13.5–16.1				
*p* value**	<0.001		<0.001				
SF-12 PCS							
Pre-operative	29.9	8.0	28.9	8.3	0.9	−1.1 to 2.7	ns
Post-operative	38.3	11.2	38.7	11.4	0.5	−2.1 to 3.2	ns
Difference	8.4	10.7	9.8	10.5	1.4	−2.6 to 3.2	ns
95% CI	6.5 to 10.3		8.3 to 11.4				
*p* value**	<0.001		<0.001				
SF-12 MCS							
Pre-operative	49.4	12.3	51.0	11.2	*1.7*	−1.0 to 4.4	ns
Post-operative	50.1	10.9	51.4	10.4	1.4	−1.1 to 3.9	ns
Difference	0.7	12.2	0.4	12.4	0.4	−1.7 to 4.0	ns
95% CI	−1.2 to 2.5		−1.2 to 2.0				
*p* value**	ns		ns				

*ASM* articular surface mounted, *CI* confidence interval, *OKS* Oxford knee score, *SF* short form, *PCS* physical component summary, *MCS* mental component summary

*t test

**Paired *t* test

There was no significant difference in the OKS between outliers (*n* = 24) and those within 3° of neutral alignment (95% CI −2.3 to 3.3, ns). However, there was a greater rate of dissatisfaction in outliers at one year but this was not statistically significant [33% (*n* = 8/24) versus 17% (*n* = 46/264), OR 2.4, 95% CI 1.0–5.9, ns].

The mean surgical time for the ASM group was 64 (SD 10) minutes and 59 (SD 11) for the control group (95% CI −2 to 12, ns). The median length of stay was 5 days (inter quartile range 4–7). There was a no significant difference in the length of stay between the ASM and control groups (ns). There were no complications from pin sites used to anchor the trackers.

## Discussion

The most important finding of the present study was that ASM navigation is significantly more accurate at achieving the desired component alignment with in a tolerance of 3 degrees compared to conventional non-navigated TKA, with an overall outlier rate of 4% compared to 11%, respectively. Interestingly, this did not affect the overall functional outcome, according to the OKS, or the rate of patient satisfaction.

Two recent meta-analyses demonstrated conflicting conclusions, with one finding no difference in outlier rate [29]and the other finding a significant improvement in component alignment and clinical outcomes for navigated TKA [30]. Registry data support the use of navigated TKA in patients less than 65 years of age, with a significantly lower revision rate when compared to conventional TKA [31]. In contrast Parratte et al. [17] demonstrated that patients with a neutral mechanical axis did not have an improved survival rate at 15 years. Despite the conflicting evidence, it is generally accepted that a neutral mechanical axis remains the gold standard [17, 32]. If this is the accepted standard it would appear that computer navigation is a more reliable tool to achieve a neutral mechanical axis. There are, however, recognised surgeon errors in alignment with navigation due to variation in bone cuts [33] and cementing techniques [34, 35].

**Fig. 2:**
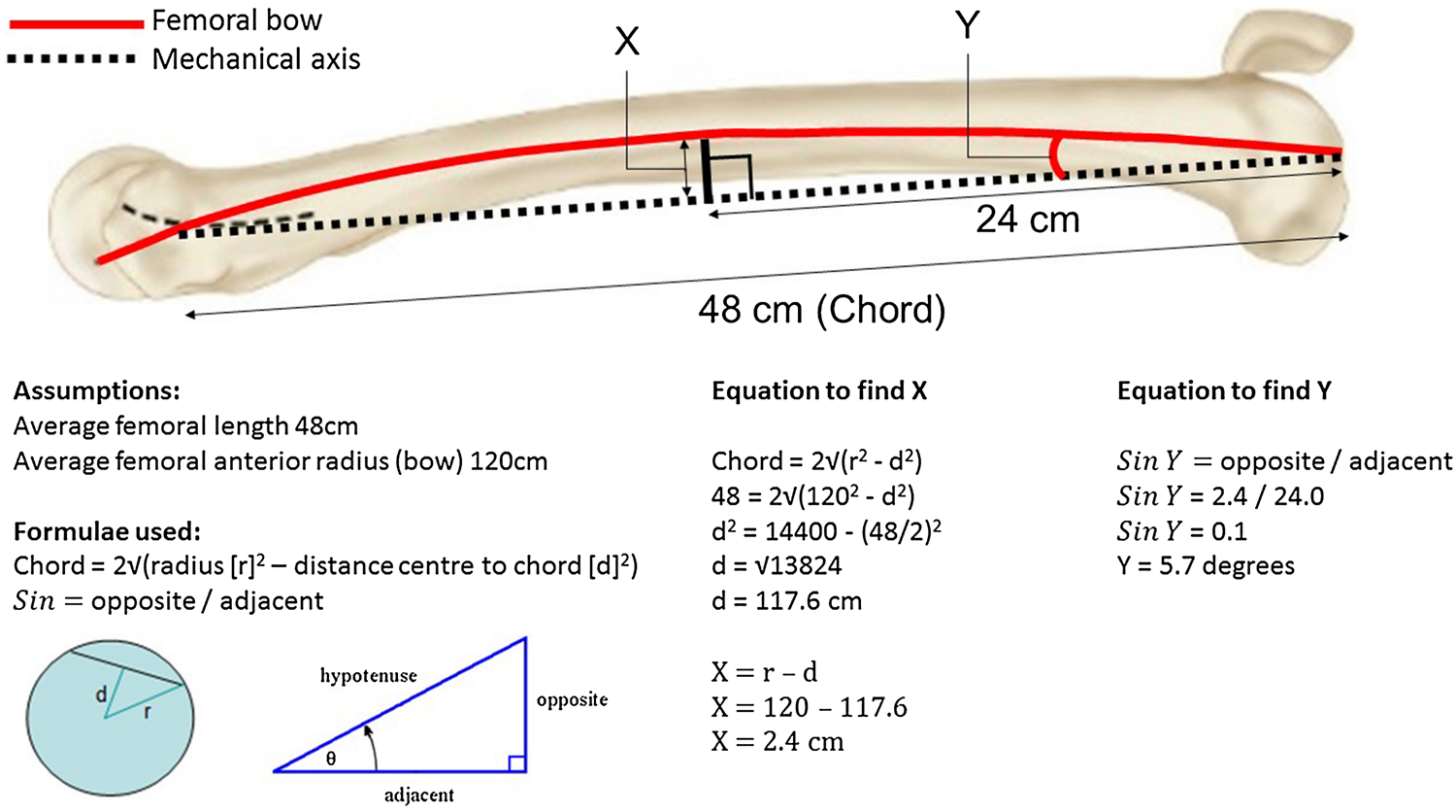
Estimation of degree for femoral flexion, relative to the mechanical axis of the femur required to compensate for anterior femoral bow

Ko et al. [36] demonstrated a significant difference in femoral flexion between navigated and non-navigated TKA, finding navigated femoral components to be extended relative to non-navigated components. Our study affirms these findings with a 3° difference between the ASM and control group. This difference may be explained by the effect of the anterior femoral bow, where an intra-medullary jig uses the bow of the distal femur to reference the flexion/extension preparation whereas the navigation uses the mechanical axis in the sagittal plane (Fig. 2). If is it is assumed on average the radius of the femur is 120 cm and the average male femur is 48 cm in length [16], using trigonometry the distal femoral component should be in 6° of flexion relative to the mechanical axis to avoid notching the distal femur (Fig. 2). This would, however, change according to the patient characteristics. Although the results from the current study suggest that extension of the femoral component does not influence the functional outcome, this could result in femoral oversizing which is associated with knee stiffness [37].

A major limitation of this study was the use of short leg radiographs to assess component alignment and the tibiofemoral angle. Ethically, it was not indicated to obtain a HKA for all patients and hence is an unavoidable limitation. The circle method used to measure each angle has previously been shown to demonstrate good correlation to those obtained from HKA measurements [24]. The second major limitation was that patients were not prospectively randomised into groups, being dependant on the availability of the ASM trays on the day of surgery. The single surgeon nature of this study is also a limitation, and the results may reflect surgical practice and not the technique. Conversely the consistency of a single surgical approach may be a positive aspect of the study, rather than comparing one surgeon performing ASM with another surgeon using a non-navigation technique as previously described [19].

If the surgeon wants to reduce their number of alignment outliers, then ASM navigated TKA should be considered as a potential tool that improves the reliability of component alignment.

## Conclusion

ASM navigation offers improved accuracy in the placement of both the femoral and tibial components when compared to conventional non-navigation alignment. However, the short-term functional outcome is not influenced by the surgical technique used.
